# Acylcarnitine profiling by low-resolution LC-MS

**DOI:** 10.1371/journal.pone.0221342

**Published:** 2019-08-15

**Authors:** David Meierhofer

**Affiliations:** Mass Spectrometry Facility, Max Planck Institute for Molecular Genetics, Berlin, Germany; University of Pittsburgh, UNITED STATES

## Abstract

Acylcarnitines are fatty acyl esters of L-carnitine and facilitate the entry of long-chain fatty acids into mitochondria via the carnitine shuttle, where they are metabolized via ß-oxidation. Alterations of acylcarnitine species can be diagnostic for fatty acid oxidation disorders and organic aciduria and are thus frequently used to screen newborns. Only a subfraction of all known acylcarnitines is thereby monitored and quantified. Therefore, a method for the simultaneous fast and robust detection of all known acylcarnitines was developed using a single concise liquid chromatography mass spectrometry (LC-MS) approach. Derivatization by 3-nitrophenylhydrazine increased the signal intensity of the acylcarnitines and a linear elution from a reversed phase column was observed that was dependent on the length of the carbon chain. This allowed a precise prediction of the exact elution time for each acylcarnitine class, which depended solely on the chemical nature of the carbon chain. This method can be further used to screen for yet unknown acylcarnitine species and adds a layer of confidence for their correct identification. Altogether 123 acylcarnitines species were used to establish a targeted low-resolution LC-MS method. The method was applied to acylcarnitine profiling in several mouse tissues and fluids, in order to identify large differences in the quantity and composition of acylcarnitines.

## Introduction

Acyl-L-carnitines are acetylated forms of L-carnitine derived from the breakdown of amino and fatty acids [1]. The acetyl group is thereby transferred from acetyl-CoA to carnitine, resulting in CoA and acylcarnitines, which play a pivotal role in facilitating the movement of acetyl-CoA into the matrices of mitochondria during the oxidation of fatty acids. Gene mutations in proteins involved in these biochemical processes can lead to alterations in the acylcarnitine profile, indicative of fatty acid oxidation and organic acid metabolism disorders. The most common of these alterations is the accumulation of the C2-C18 acyl-CoA species, which are substrates for one of several carnitine acyl-CoA transferases [2]. Many gene mutations are directly associated with specific increases or decreases in acylcarnitine species, as summarized in several reviews [2, 3]. Urine, plasma, blood or dried blood spots are easy to access for screening and are hence the sources of choice for analyzing acylcarnitine deficiencies. A one-dimensional separation of acylcarnitines is commonly done by reversed phase [4–6] or HILIC chromatography [6, 7]. Though a high number of identified molecular species have previously been demonstrated by high-resolution instruments [5–7], quantitative data for comparative studies are only available for a limited number of compounds.

The goal of this study was to establish comprehensive LC-MS based methods to screen all known acylcarnitines, by using a low-resolution (QTrap) mass spectrometer to overcome the current limitations in the number of quantifiable compounds. Thus, all available acylcarnitine entries were extracted from the METLIN [8] database and the Human Metabolome Database (HMDB) [9], and a recent publication [6]. Acylcarnitines were derivatized by 3-nitrophenylhydrazine (3NPH), which modifies all carboxyl groups of acylcarnitines [10]. By adding a isotope-labeled 3NPH reference sample, a direct and unbiased comparison of two or more conditions is possible, since an identical metabolite extraction work-flow and LC-MS conditions are applied [11], but were not used in the current study. Optimized multiple reaction monitoring (MRM) parameters for all acylcarnitine species were applied in combination with a chromatographic separation on a reverse phase (C18) column to create a scheduled MRM method. The carbon chains of the acylcarnitines were thereby separated according to their carbon chain length, saturation state, and the presence of hydroxyl- and di-carboxyl groups. These features were used to plot acylcarnitines according to their molecular mass and specific retention time, resulting in a linear assembly of each chemically distinct class. This adds a level of confidence for the identification of compounds and enables the prediction of the retention time for yet unknown acylcarnitines, solely based on their chemical nature. Acylcarnitine profiles of five different mouse tissues and two fluids were compared in this study. Hence, the method presented allows the rapid and confident identification and quantification of acylcarnitines using a low-resolution LC-MS device.

## Materials and methods

### Reagents

Formic acid, ammonium acetate, and 3-nitrophenylhydrazine (#N21804) were acquired from Sigma-Aldrich (St. Louis, MO), 1-ethyl-3-(3-dimethylaminopropyl)carbodiimide (EDC, #2156.1) and pyridine (#CP07.1, 99%) were from Roth (Karlsruhe, Germany). Authentic acylcarnitine standards were obtained from Cambridge Isotope Laboratories (Andover, MA, #NSK-B), and Sigma-Aldrich (2-methylbutyryl-L-carnitine #50405, isovaleryl-L-carnitine #51371, valeryl-L-carnitine #04265, oleoyl-L-carnitine (C18:1) #19945, and O-succinyl-L-carnitine (C4-DC) #04609. All other reagents were of analytical LC-MS grades.

### Mouse tissues and fluids

Animals were obtained from ENVIGO (Huntingdon, United Kingdom), transferred into our Specific-Pathogen-Free facility and provided with food and water *ad libitum*. For tissue collection, two 46-week-old *wt* C57 BL5/7 mice were sacrificed by cervical dislocation. The following tissues were dissected and fluids were taken within minutes and immediately stored at -80°C: brain, heart, liver, visceral fat, thigh muscle, as well as whole blood and serum. All experimental procedures were approved by the governmental authority (Landesamt für Gesundheit und Soziales, LAGeSo, Berlin, Germany). All animal experimental procedures were carried out in accordance with the approved guidelines of the LAGeSo.

### Metabolite extraction and derivatization of acylcarnitine by 3NPH

Five mg of frozen target tissues, 17 μL whole blood, and 8 μL plasma were used to extract metabolites in biological triplicates. One mL of 80/20 methanol/water was added, and the tissues were additionally homogenized with a FastPrep (one time for 60 s, 4.5 m x s^-1^). Debris was removed by a 10 min centrifugation at 20,000 rcf at 4°C and the supernatants were aliquoted. An isotopically labeled internal acylcarnitine standard (IS, 20 pmol), containing 8 acylcarnitines species was added. Derivatization of acylcarnitines by 3-nitrophenylhydrazine (3NPH) was performed as previously reported [10]. In brief, 25 mM 3NPH (5 μL of 0.5 M in 35% acetonitrile), 25 mM EDC (2.5 μL of 1M in water), and 0.396% (0.4 μL, 99%) pyridine were added to the samples (in 80% methanol) sequentially and kept for 30 minutes at 30°C on a rocking platform. Samples were lyophilized and dissolved in 30 μL water before usage and 15 μL were injected. A 100 pmol aliquot of the IS was further used to validate the derivatization efficiency by comparing the ratios between derivatized and non-derivatized IS.

### Acylcarnitine screening by low-resolution LC-MS

The three isomeric acylcarnitine standards (2-methylbutyroylcarnitine, isovalerylcarnitine, and valerylcarnitine), oleoyl-L-carnitine (C18:1), O-succinyl-L-carnitine (C4-DC), and an isotopically labeled standard consisting of eight acylcarnitines (C0-C16) were derivatized by 3NPH. The automatic optimization procedure in the Analyst software (v.1.6.2) was used to identify and optimize the collision energy and the declustering potential of the most intense fragments.

Subsequent separation of standards was performed on an LC instrument (1290 series UHPLC; Agilent, Santa Clara, CA), online coupled to a triple quadrupole hybrid ion trap mass spectrometer (QTrap 6500, Sciex, Foster City, CA), as reported previously [12]. Two different LC columns and several different buffer conditions were used to identify the highest peak areas, optimal peak shapes, and linear retention times within acylcarnitine classes, as described previously [12]. Finally, a Reprosil-PUR C18-AQ (1.9 μm, 120 Å, 150 x 2 mm ID; Dr. Maisch; Ammerbuch, Germany) with the following buffer and run conditions were selected for metabolite separation: A1: LC-MS grade water; 0.1% formic acid; B1: LC-MS grade acetonitrile; 0.1% formic acid. Gradients and flow conditions were as follows: the compounds were separated by a linear increase of B1 from the first minute from 2% to 100% in 36 minutes. The flow rate was reduced from 275 to 225 μL min^-1^ at the same time. These conditions were kept for 1 minute and followed by a reduction to 2% B1 within 1 minute. The equilibration of the column continued until minute 44, while the flow rate was maintained at 275 μL min^-1^. The three most common and intense fragments (220, 145, and 84 Da) were selected (except for C0 carnitines).

Metabolite identification was based on three levels: (i) the correct retention time, (ii) the three common transitions for all acylcarnitines (iii) and a matching MRM ion ratio of tuned pure metabolites used as a reference [12]. Relative quantification was performed with MultiQuantTM software v.2.1.1 (Sciex, Foster City, CA). The integration setting was a peak splitting factor of 2 and all peaks were reviewed manually. Only the peak area of the first transition was used for quantification. Normalization was done according to the used wet weight of tissues or the volume of the fluids and subsequently by the derivatized ^13^C acylcarnitine standard. All transition settings for the targeted MRM method are provided in S1 Table.

To assign identified acylcarnitine fragment masses to structural information, the high-resolution LC-MS instrument Q-Exactive HF Orbitrap mass spectrometer (Thermo Scientific, Waltham, MA) was used for recording precise MS^2^ spectra, which were finally mapped to structures by applying the ACD Spectrus Processor 2017.2.1 software. In brief, LC-MS was carried out by nanoflow reverse phase liquid chromatography (Dionex Ultimate 3000, Thermo Scientific,) coupled online to a Q-Exactive HF Orbitrap mass spectrometer. The LC separation was performed using a PicoFrit analytical column (75 μm ID × 55 cm long, 15 μm Tip ID, New Objectives, Woburn, MA) in-house packed with 3-μm C18 resin (Reprosil-AQ Pur, Dr. Maisch, Ammerbuch-Entringen, Germany). Metabolites were eluted using a gradient from 12 to 95% solvent B in solvent A over 50 min at 266 nL per minute flow rate. Solvent A was 0.1% formic acid and solvent B was 79.9% acetonitrile, 20% H_2_O, 0.1% formic acid. Nanoelectrospray was generated by applying 3.5 kV. A cycle of one full Fourier transformation scan mass spectrum (50–700 m/z, resolution of 30,000 at m/z 200, AGC target 5e5) was followed by a parallel reaction monitoring (PRM) scan (MS/MS scan, resolution of 30,000, AGC target 1e5, max IT 100 ms, isolation window of 0.6 m/z) with a stepped collision energy of 25, 35, and 50 eV for positively and single charged metabolites.

### Statistical analysis

Biological triplicates from all tissues and fluids were analyzed. A two-sample t-test was performed and volcano plots were generated using Perseus (v1.6.1.3) [13].

## Results and discussion

This study applied 3NPH derivatization of acylcarnitines for comprehensive identification, as shown in a low-resolution quadrupole mass spectrometer. Derivatization by 3NPH [10] was used to increase the sensitivity and most importantly, to achieve a linear elution profile on a reversed phase column for all acylcarnitine classes, which is not the case for short acylcarnitines without derivatization [4].

**Fig 1:**
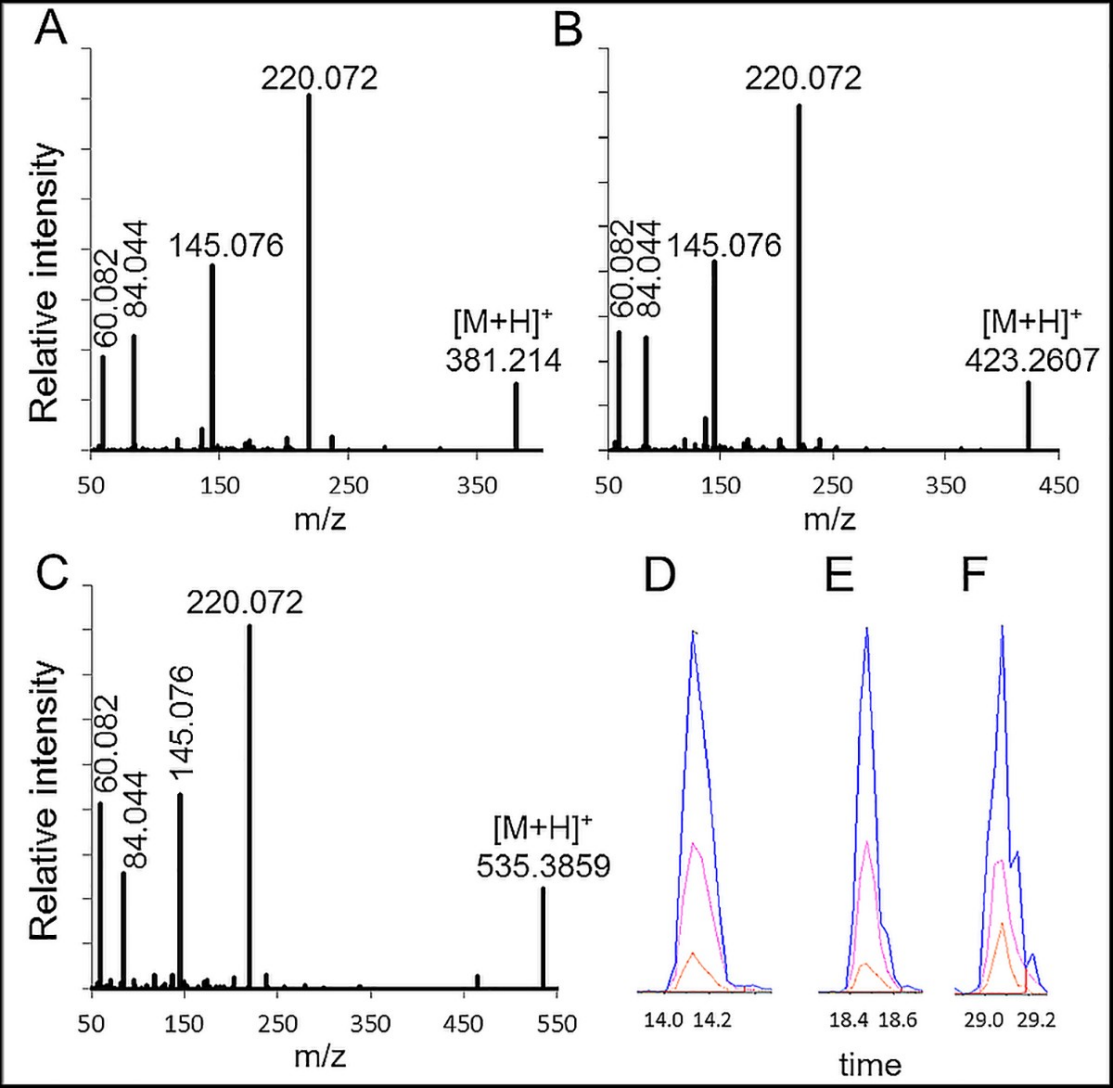
High-resolution fragment spectra (A-C) and low-resolution transitions (D-F) of 3NPH derivatized acylcarnitines. MS^2^ spectra of the endogenous 3NPH acylcarnitines: (A) valerylcarnitine (C5:0), (B) octanoylcarnitine (C8:0), and (C) palmitoylcarnitine (C16:0). The three most intense transitions (220, 145, and 84 Da) of the same metabolite species, but isotopically labeled, were monitored by a low-resolution QTrap instrument: (D) isovalerylcarnitine-D9 (C5:0, 390.43 Da, RT 14.14), (E) octanoylcarnitine-D3 (C8:0, 426.47 Da, RT 18.47), and (F) palmitoylcarnitine-D3 (C16:0, 538.69 Da, RT 29.07). Following fragment masses were monitored: 220.1 (blue); 145.1 (red) and 84 Da (orange). No Gaussian smoothing was used.

Derivatized standards were used to identify the three most abundant transitions, which were the fragment masses of 220, 145, and 84 Da (Fig 1). The only exception was found for C0 acylcarnitines (S1 Table).

**Fig 2:**
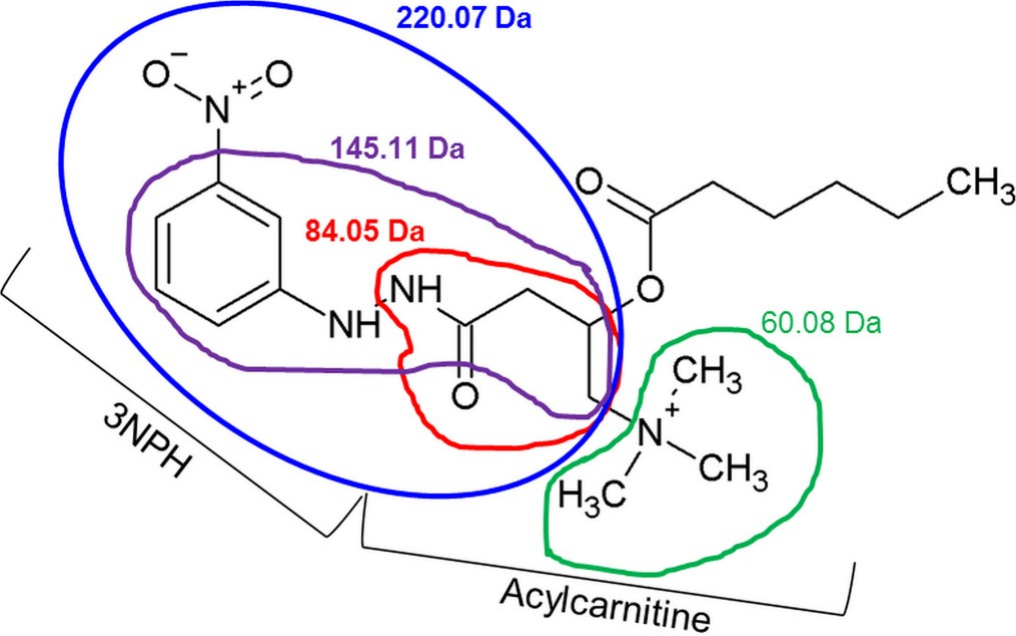
Chemical structure and identified fragment ions generated in the collision cell of the mass spectrometer of a C6:0 acylcarnitine, derivatized by 3NPH. Displayed are the four most common fragments found in all 3NPH-acylcarnitines.

A high-resolution Orbitrap instrument and the software ACD Spectrus Processor were utilized to assign fragment masses to structures, differing ≤ 0.001 Da from the theoretical masses. For example, the 220 Da fragment was always the most intense ion and included the 3NPH molecule, the 60 Da fragment ion represented the trimethylamine ion (Fig 2).

### Derivatization by 3NPH

To evaluate, whether the derivatization process for acylcarnitines by 3NPH was complete, the isotope standard, containing eight different deuterium labeled acylcarnitine species of varying concentrations, was prepared and derivatized as described in the methods. A 100 pmol aliquot of the IS was loaded onto the LC-MS in triplicate. Optimized transition settings for derivatized and non-derivatized standards were used to monitor the peak areas in a single LC-MS run in parallel. Virtually no peaks were found for the non-derivatized acylcarnitines. The mean peak areas of the first transitions were only 0.075% compared to the derivatized peak areas. Hence, the applied method achieved full derivatization (S2 Table). Based on the isotopically labeled standard, the peak area of the first transition increased on average by 1.8-fold versus the unlabeled acylcarnitines (S3 Table). Thus, the derivatization of carboxy groups by 3NPH is fast, robust, and reliable, as shown in the original report [10].

All available acylcarnitine entries from the two databases, METLIN [8] and the Human Metabolome Database (HMDB) [9],and a recent publication [6] were extracted to build an MRM database. In addition, acylcarnitine species, which might exist, were added, resulting in a list of 123 molecular acylcarnitine species. Transitions were thus generated based on the three most common previously identified fragment ions 220, 145, and 84 Da. MRM settings, such as the declustering potential (DP) and the collision energy (CE) were optimized by utilizing a liver sample for all acylcarnitine species. Whenever signal intensities were too low, settings from the same acylcarnitine class with a similar carbon chain length were used (S1 Table).

### Linear elution profiles of 3NPH derivatized acylcarnitines classes according to chemical characteristics

**Fig 3:**
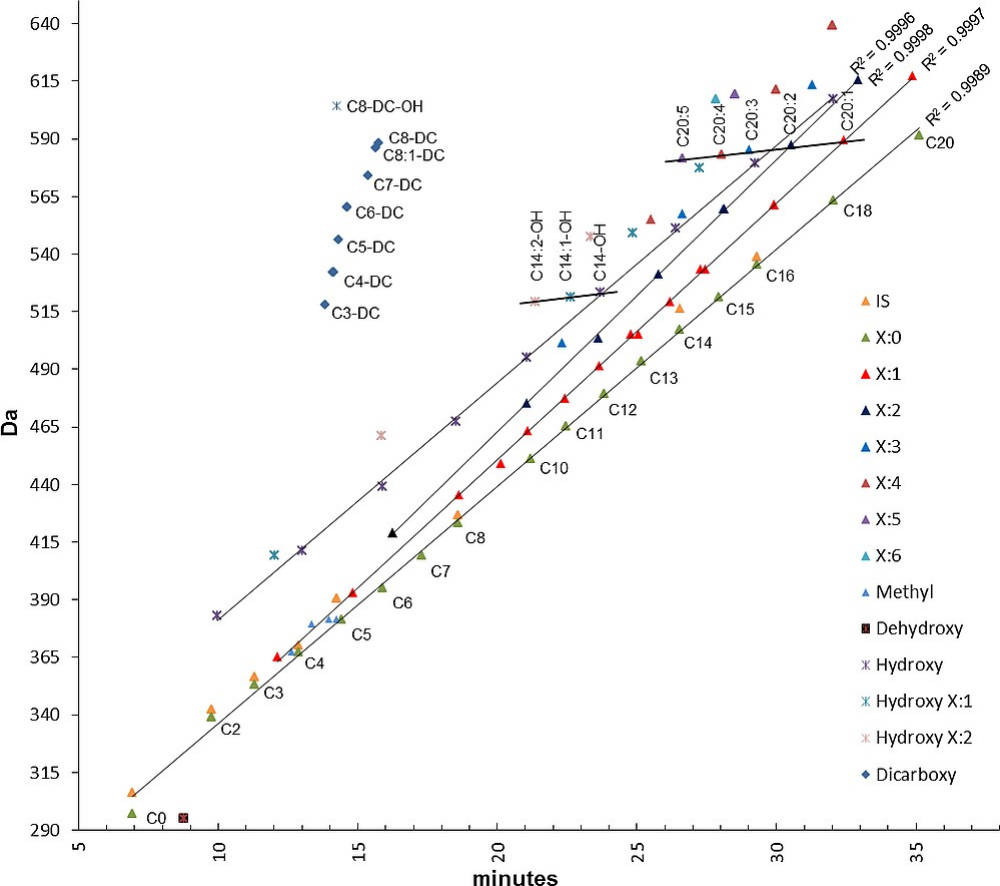
A mass versus retention time plot of all repeatedly identified acylcarnitines (shown for liver) shows a linear regression, R^2^ values close to 1, for each acylcarnitine class. An increasing carbon number of the carbon chain shifts the elution time to the right, leading to a straight line of saturated acylcarnitines in the plot (green triangles). Carbon chains containing hydroxyl groups shifted in a parallel way to the left (purple cross), unsaturated species (red, black, and blue triangles) also to the left, but more apparent for longer carbon chain types. All dicarboxylic containing species eluted at very similar time points (blue diamonds), isotopically labeled internal standards are shown in orange triangles.

The elution order in a reversed phase system is governed by the water solubility and carbon content of the molecule [14, 15]. Therefore, the retention time increases as the number of carbon atoms increases. All acylcarnitine classes thus eluted linearly in a Da versus retention time plot, starting from C0 to C20 (Fig 3).

Chemical variations, which determine an acylcarnitine class, increased or decreased the elution time from the C18 column, but the linearity of the entire class was always observed (Fig 3). Unsaturated acylcarnitines, for example, are more polar due to the π electron dipole in the double bond and hence elute earlier. Branched-chain compounds also elute more rapidly than normal isomers and hydroxylated species show the least retention, whereas dehydroxylation leads to a delayed elution time. Acylcarnitines, which feature two carboxy groups, are derivatized in two places and hence elute in a separate cluster (Fig 3). These unique elution patterns spanned the entire range from C0 to C22 with an R^2^ close to 1 and thus can be used to better identify every compound class. In addition, transitions for so far unknown acylcarnitines can be calculated and the exact retention time can be precisely predicted by the linear regression curve. For example, a C19 acylcarnitine has the mass of 577.4 Da and would hit the liner regression curve at 33.9 minutes. This is an advantage, since there are currently almost no commercially available standards for the majority of acylcarnitines, which would need to be synthesized individually. The knowledge of the exact retention time, specifically for predicted compounds, is thus crucial for comprehensive identification, since the vast majority of erroneous peaks can be excluded.

The 3NPH derivatization of acylcarnitines has the advantage over a previous method [4] that also identified short-chain acylcarnitines of each class eluted in a linear way. This derivatization enables a simple determination of the precise elution time for all unknown acylcarnitines in the same class: The elution time is the point where the calculated mass cuts across the linear regression line for a specific class (Fig 3). Since a different compound class shifts the time scale, only a few standards are necessary to determine the linear gradient. Only the unsaturated compounds follow a slightly different equation, because long unsaturated compounds decrease their retention time on the column.

### Distinguishing isomeric acylcarnitines

**Fig 4:**
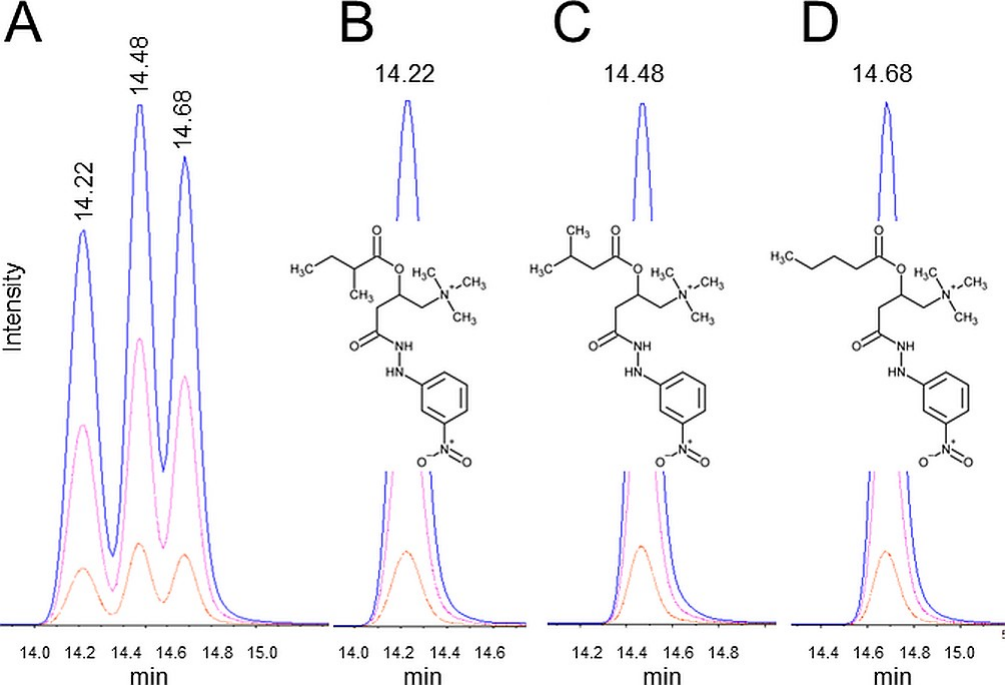
Identification of isomeric acylcarnitines. (A) Shown are three isomeric metabolites in one combined LC-MS run and individually: (B) 2-methylbutyroylcarnitine (C4-methyl), (C) isovalerylcarnitine (C4-methyl), and (D) valerylcarnitine (C5). Following transitions were used: 381.2 / 220.1 (blue); 381.2 / 145.1 (red) and 381.2 / 84 Da (orange), a Gaussian smooth width of 2 was used.

Isomeric acylcarnitines have the same number of atoms of each element, but have a different arrangement of atoms in the carbon chain. Unfortunately, fragments of the carbon chain are of low intensity and thus cannot always be used to identify the acylcarnitine class. Hence, isomeric acylcarnitines can be separated using LC, since each acylcarnitine class elutes slightly differently. For example, there are four isomeric acylcarnitines with a mass of 381.21379 Da (non-derivatized 245.16271), namely valerylcarnitine (C5), isovalerylcarnitine (C4-methyl), 2-methylbutyroylcarnitine (C4-methyl), and pivaloylcarnitine (C3-dimethyl). Three peaks were always found in endogenous samples by monitoring the identical transitions of these four isomeric acylcarnitines. To elucidate the identities of all peaks, three commercially available standards (not available for pivaloylcarnitine) were employed (Fig 4A). The first peak eluted was 2-methylbutyroylcarnitine at minute 14.22 (Fig 4B), followed by isovalerylcarnitine in the middle at minute 14.48 (Fig 4C) and valerylcarnitine was eluted last at minute 14.68 (Fig 4D). The elution profile is in accordance with the rule that branched carbon chains elute first. Pivaloylcarnitine was expected to elute as the very first compound, but no endogenous peak was identified, indicating that this species was not present in the samples. Thus, also isomeric acylcarnitines can be distinguished solely based on differences in their elution times, where the methyl groups decrease their affinity for the column and therefore elute earlier.

The localization complexity of C = C bonds increases with the length of the carbon chain. For many such acylcarnitines, we detected multiple peaks in an organ-specific manner, but could not assign the peaks to the position of the double bond, since the corresponding standards were not available and double bonds do not generate specific fragments. Furthermore, the three-dimensional structure might also have an influence on the retention time, especially for long chain and multiple unsaturated species. But since the biochemical and biophysical properties of lipids are also determined by the positions of their double bonds, their identification is of interest. The chemistry of the Paternò–Büchi reaction towards the C = C double bond can be used for lipid structural characterizations[16, 17]. Recently, an online coupling of the Paternò–Büchi reactions with mass spectrometry showed the formation of diagnostic ions specific to the double bond location within unsaturated lipids[18]. This strategy preserves the unique linear elution profiles for 3NPH-acylcarnitines and simultaneously identifies the precise position of the double bonds, which can be monitored by additional transitions.

Taken together, the presented targeted low-resolution mass-spectrometry method excels in the robust identification of acylcarnitines: Repeatedly identified compounds reflect the known acylcarnitines within the databases Metlin and HMDB and the mass versus RT plot shows linearity for metabolites of all classes in a single LC-MS run and thus eliminates the majority of false hits. Modern mass spectrometers can record mass spectra every other millisecond, resulting in a myriad of spectra per run. Without strict and appropriate data filtering, the user drowns in false identifications. Furthermore, QTrap mass spectrometers are the most sensitive instruments available. A general topic in the field of metabolomics and lipidomics is the lacking availability of standards, specifically for isotope-labeled standards, which hampers the simple identification of spectra.

### Acylcarnitine profiling in mouse tissues and fluids

Acylcarnitine species are released or taken up in a dynamic and organ-specific way, that is dependent on the demand for ATP [19]. Furthermore, dysregulation of specific acylcarnitine species is recognized as being important in the pathophysiology of obesity and has emerged as a risk factor for the development of diabetes [20–22]. Hence, acylcarnitine profiles for five main mouse tissue types (brain, heart, liver, visceral fat, thigh muscle) and two fluids (whole blood and serum) were monitored with the established method to elucidate the distribution and quantity of acylcarnitine species.

The number of acylcarnitine species identified within distinct mouse tissues and fluids varied, the highest number was found in liver (90), followed by the heart (87), visceral fat (65), thigh muscle (62), serum (62), whole blood (58), and brain (57, S4 Table). The most abundant molecular species in all tissues were acetylcarnitine, carnitine, 3-dehydrocarnitine, and the C3, C3-methyl, C4, C4-OH, C6, C16, C16:1, C18, C18:1 and C18:2 species (S1 Fig).

**Fig 5:**
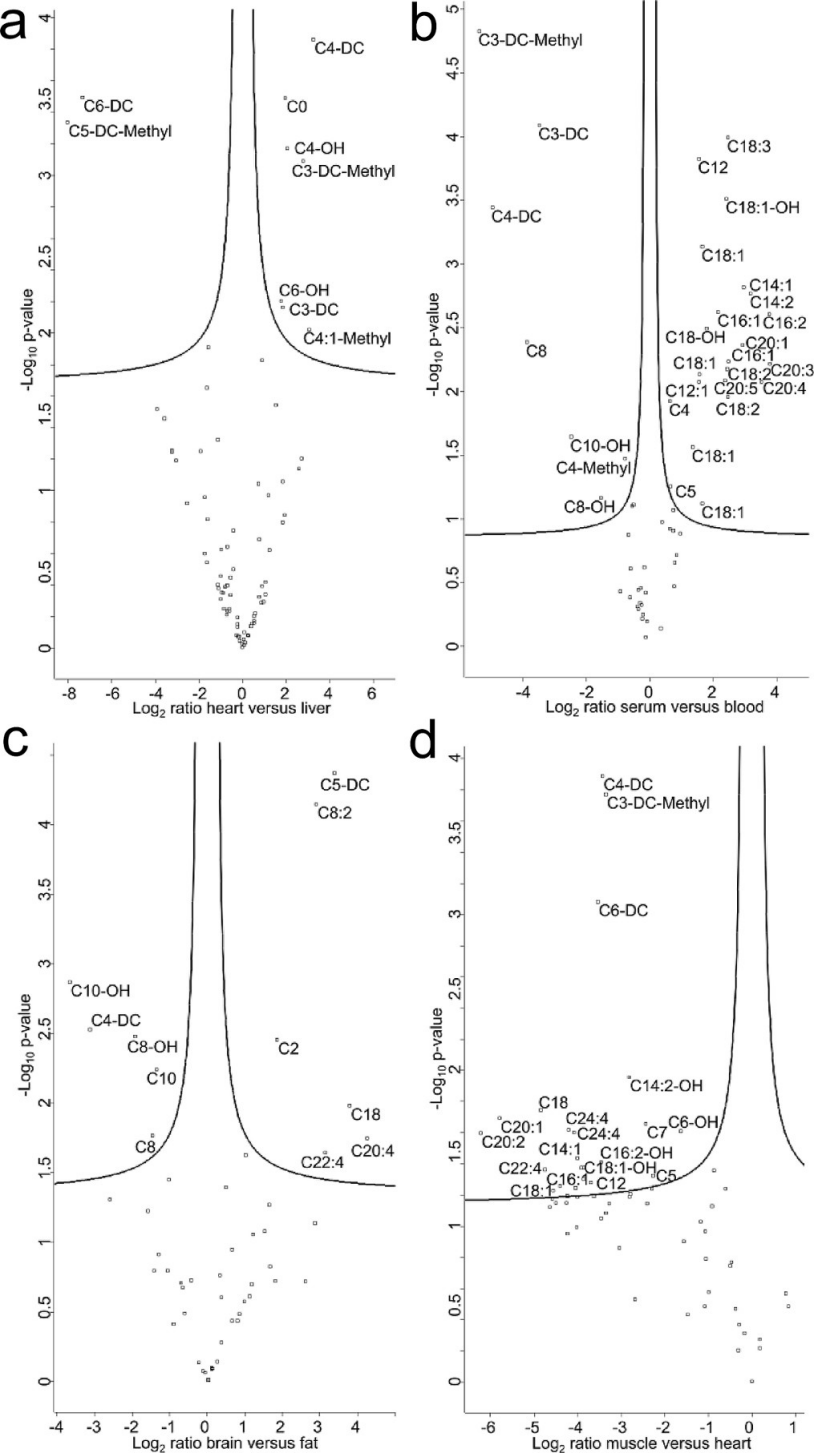
**Volcano plots of acylcarnitines in mouse tissues and liquids: (A) heart versus liver, (B) serum versus blood, (C) brain versus fat, (D) muscle versus heart.** The negative log_10_-transformed *p*-values of the Student's t-test are plotted against the log_2_ ratios of fold change between specimens. The solid lines represent the nominal significance level for the Student's t-test (FDR ≤ 0.05). Three biological replicates were used for each specimen.

To significantly identify altered acylcarnitines between tissues and fluids, a t-test was performed and displayed in volcano plots (Fig 5). Between heart and liver tissues, significant changes were observed for short-chain dicarboxylic acylcarnitines, which were either increased or decreased (Fig 5A). Carnitine levels were unchanged, but 3-dehydrocarnitine was significantly increased in the heart. In comparing serum versus blood, unsaturated C16, C18, and C20 acylcarnitine species were found to be significantly enriched in serum and several dicarboxylic- and short-chain acylcarnitines were increased in the blood (Fig 5B). Thigh muscle tissue has, in general, less abundant acylcarnitines compared with heart tissue; significant reductions were seen in unsaturated and hydroxylated long-chain acylcarnitines and short chain dicarboxylic compounds (Fig 5D). All log_2_ transformed peak areas are shown in S4 Table.

### Conclusions

The low-resolution LC-MS method described here enables the fast and reliable identification and quantification of even and odd numbered as well as isomeric forms of 3NPH derivatized acylcarnitines. Comprehensive identification of these derivatized acylcarnitines, including hypothetical acylcarnitine species, can be achieved by calculating linear regression curves for each class in a mass versus retention time plot. Linearity was also seen for short-chain acylcarnitines, which was not the case in a previous analysis without derivatization [4]. The scheduled MRM method includes eight isotopically labeled internal standards and 123 acylcarnitine species with a total of 393 transitions. The same methodology can also be applied for high-resolution mass spectrometers, such as TOF and OrbiTrap instruments. In this way, a parallel reaction monitoring (PRM) method can be used, which additionally allows the verification of the identified compounds by their precise masses and MS^2^ spectra, as shown recently for acylcarnitines [4]. The targeted LC-MS method presented here has the advantage of being highly reliable since it utilizes three tuned transitions per acylcarnitine species and a precisely predictable retention time due to the linear regression for each acylcarnitine class in a single LC-MS run.

## Supporting information

S1 Fig Log_2_ abundances of acylcarnitines. Acylcarnitine profiling in mouse serum, blood, muscle, fat, liver, heart, and brain. Average log_2_ peak areas of three biological replicates.
(TIF)

S1 Table List of all acylcarnitine transitions and MRM instrument settings for the low-resolution LC-MS method. Acylcarnitine names, chemical formulas (before derivatization), Metlin and HMDB identifiers, Q1 and Q3 masses, MRM settings, MRM ion ratios and used internal standards are provided. DP = declustering potential; CE = collision energy; CXP = collision exit potential.
(XLSX)

S2 Table 3NPH derivatization efficiency. (XLSX)

S3 Table Comparison of derivatized versus non-derivatized isotope labeled standard, 3 replicates. (XLSX)

S4 Table Log_2_ abundances (peak areas) of all mouse tissues and fluids in biological triplicates. The first transition is shown and was used to calculate ratios and p-values; n.d., not determined, if not present in all replicates of a sample.
(XLSX)

## Abbreviations

LC-MS: Liquid chromatography–mass spectrometry
3NPH: 3-nitrophenylhydrazine
MRM: multiple reaction monitoring
