# Supplementary figures and images for: Acylcarnitine profiling by low-resolution LC-MS
Source: PLoS One. 2019 Aug 15;14(8):e0221342. doi: 10.1371/journal.pone.0221342 (PMC6695155; doi:10.1371/journal.pone.0221342)

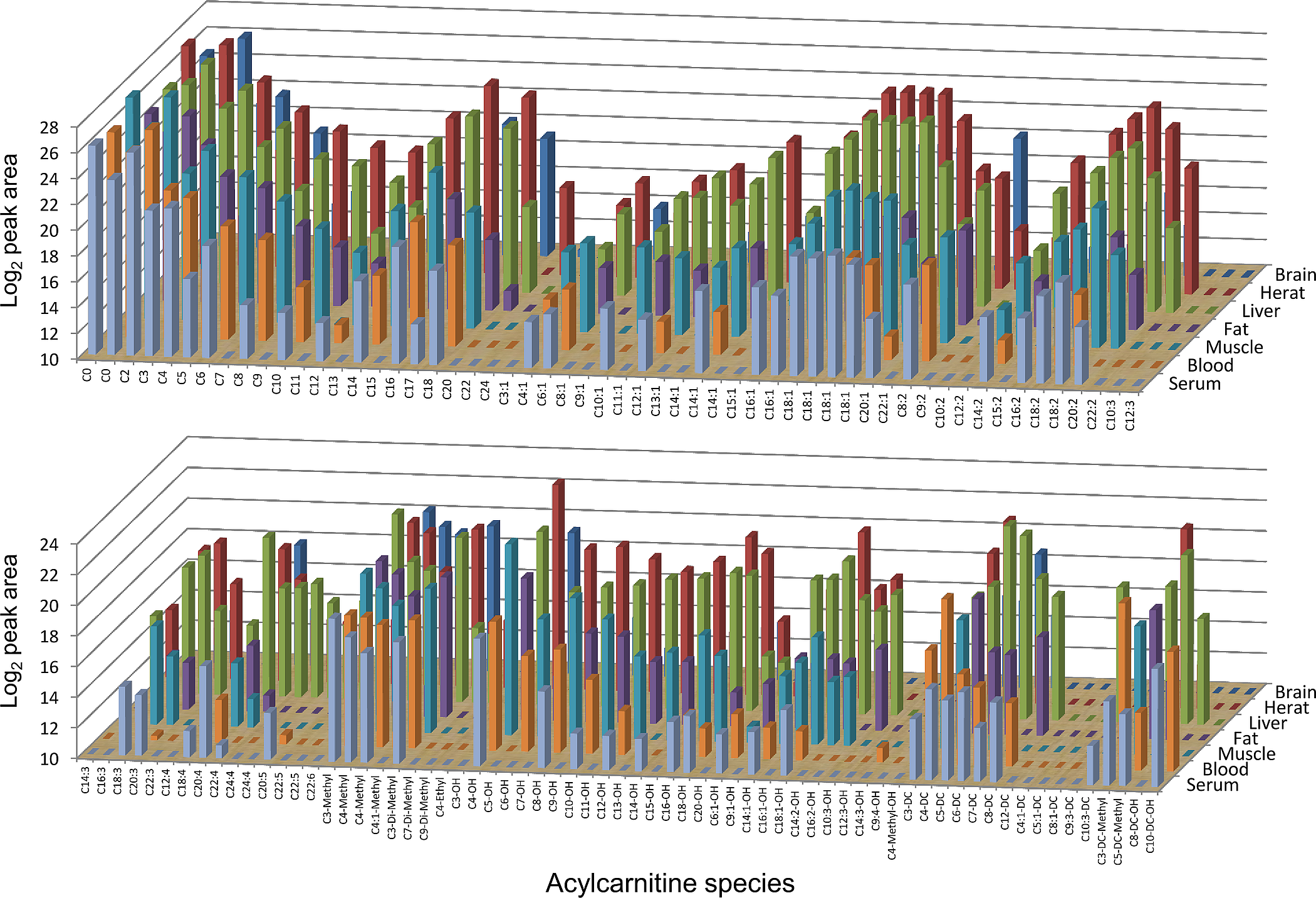

Supplement: S1 Fig — Acylcarnitine profiling in mouse serum, blood, muscle, fat, liver, heart, and brain. Average log2 peak areas of three biological replicates. (TIF) [file pone.0221342.s001.tif]
